# Brunner syndrome caused by point mutation explained by multiscale simulation of enzyme reaction

**DOI:** 10.1038/s41598-022-26296-7

**Published:** 2022-12-19

**Authors:** Alja Prah, Domen Pregeljc, Jernej Stare, Janez Mavri

**Affiliations:** 1 Theory Department, National Institute of Chemistry, Ljubljana, Slovenia; 2 Network Infrastructure Centre, Jožef Stefan Institute, Ljubljana, Slovenia; 3 Department of Chemistry, Imperial College London, London, UK

**Keywords:** Computational neuroscience, Biocatalysis

## Abstract

Brunner syndrome is a disorder characterized by intellectual disability and impulsive, aggressive behavior associated with deficient function of the monoamine oxidase A (MAO-A) enzyme. These symptoms (along with particularly high serotonin levels) have been reported in patients with two missense variants in MAO-A (p.R45W and p.E446K). Herein, we report molecular simulations of the rate-limiting step of MAO-A-catalyzed serotonin degradation for these variants. We found that the R45W mutation causes a 6000-fold slowdown of enzymatic function, whereas the E446K mutation causes a 450-fold reduction of serotonin degradation rate, both of which are practically equivalent to a gene knockout. In addition, we thoroughly compared the influence of enzyme electrostatics on the catalytic function of both the wild type MAO-A and the p.R45W variant relative to the wild type enzyme, revealing that the mutation represents a significant electrostatic perturbation that contributes to the barrier increase. Understanding genetic disorders is closely linked to understanding the associated chemical mechanisms, and our research represents a novel attempt to bridge the gap between clinical genetics and the underlying chemical physics.

## Introduction

Neuropsychiatric disorders represent a common group of complex conditions that have a significant impact on the quality of life of those affected. When speaking of neuropsychiatric disorders, one usually refers to schizophrenia, major depressive disorder, bipolar disorder or attention deficit hyperactivity disorder (ADHD)^1^. They can all be broadly characterized by changes in brain function, the specific causes of which are reflected in their corresponding neuropathologies and are usually caused by imbalances in the level of neurotransmitters, usually as a result of irregularities in their metabolism. One group of enzymes involved in the degradation of neurotransmitters are monoamine oxidases (MAOs). MAOs are enzymes that catalyze the oxidative deamination of monoaminergic neurotransmitters. The two genes coding for two MAO isoenzymes, A and B, are located on X chromosome. MAO-A and MAO-B share 70% homology but differ in their tissue distribution, inhibitor specificity and substrate specificity—MAO-A preferentially metabolizes serotonin, epinephrine, and norepinephrine, while the preferred substrates for MAO-B are phenylethylamine and benzylamine.

Brunner syndrome (OMIM #300615) was first described by H. G. Brunner et al*.* in 1993 in a large Dutch family with X-linked borderline intellectual disability and abnormal behavior (including impulsive aggression, arson, attempted rape, and exhibitionism)^2^. It was suggested that these symptoms might be related to MAO-A deficiency, and sequencing revealed a mutation in the *MAOA* structural gene in each of the five affected men. A nonsense c.886C > T (p.Q296*) mutation changes a glutamine (CAG) codon to a termination (TAG) codon at position 296 of the amino acid sequence, resulting in a truncated variant of the enzyme. In addition, urinalysis showed increased excretion of normetanephrine and tyramine and decreased concentrations of 5-hydroxyindoleacetic acid, homovanillic acid, and vanillylmandelic acid, all indicating alterations in central neurotransmitter metabolism. Later on, other missense mutations in the *MAOA* gene were found in several families and individuals sharing a similar clinical picture that included autism spectrum disorder, aggressive behavior, attention deficit disorder and intellectual disability. Examples of such mutations are p.C266F^3^, p.E446K^4^, p.R45W^5^ as well as a truncated variant p.S251KfsX2^5^. In all these cases, apart from a bold clinical picture, biochemical analysis of MAO-A substrates and metabolites in patients’ plasma and urine revealed changes in the degradation of serotonin and catecholamines (mainly norepinephrine), with particularly elevated urinary levels of metanephrine and normetanephrine. These observations provide strong evidence of a drastically altered metabolism of neurotransmitters, clearly pointing at deficient catalytic performance of the MAO-A enzyme as a possible root cause. Decreased MAO-A activity results in increased levels of serotonin already at prenatal stage giving rise to altered neuroplasticity that cannot be treated pharmacologically.

**Figure 1:**
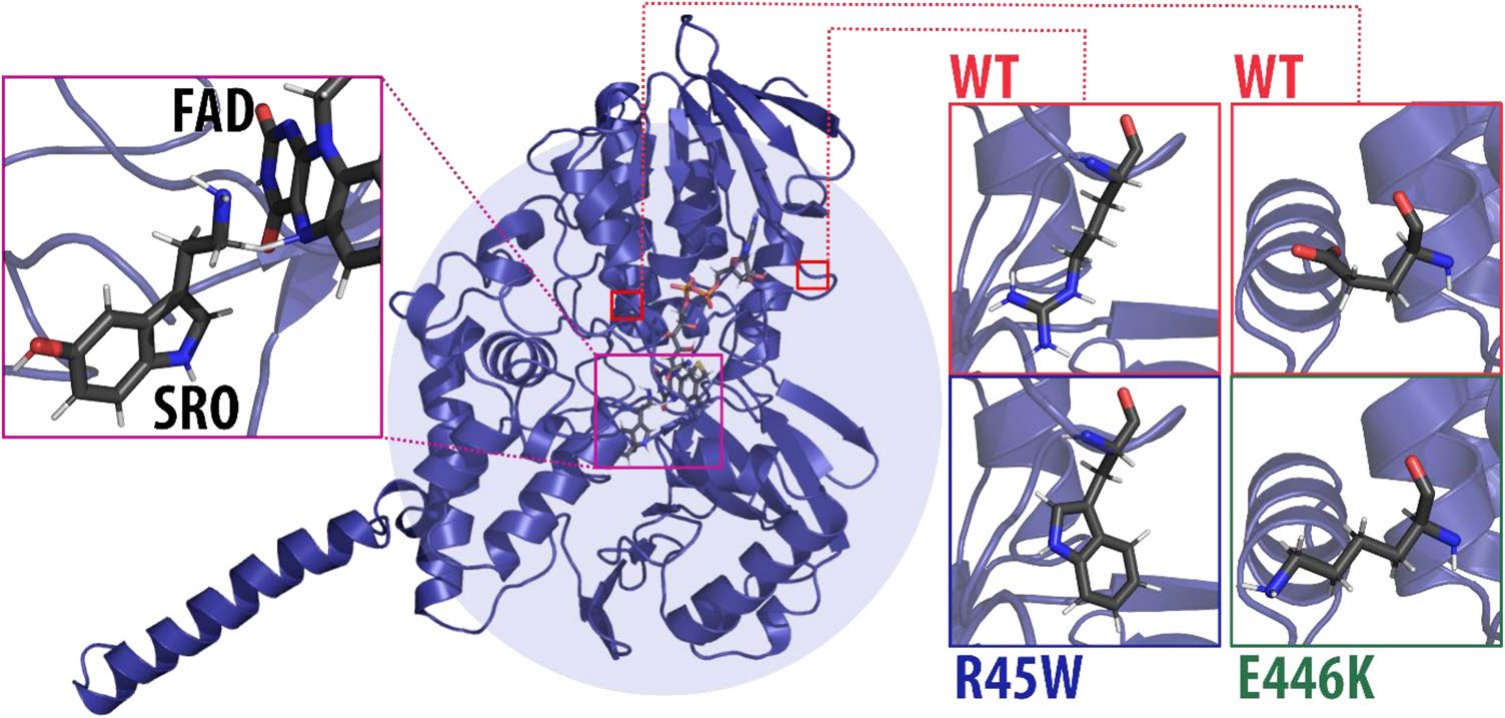
Monoamine oxidase enzyme with serotonin (SRO) docked in the active site (shown in inset on the left) and reacting with the flavin adenine dinucleotide cofactor (FAD). The locations of the mutated residue of two mutation variants (R45W and E446K) are marked with red squares. In R45W amino acid arginine from the wild type enzyme (WT) is replaced by tryptophan, whereas in E446K glutamate is replaced by lysine.

Among the aforementioned MAO-A variants, the p.R45W mutation identified by Palmer et al*.*^5^ deserves special attention, as it features a particularly prominent clinical picture with a large array of serotonergic symptoms. In this missense mutant the amino acid arginine at position 45, located in the flavin adenine dinucleotide (FAD) binding domain, is replaced by a larger and more hydrophilic tryptophan (Fig. 1). The authors suggest that this mutation affects serotonin metabolism with MAO-A more than other bioamines and that behavioral abnormalities associated with Brunner syndrome are a direct consequence of elevated serotonin levels. Similar clinical picture accompanied with elevated levels of neurotransmitters in serum and in urine is observed for the p.E446K mutation^4^, suggesting that the performance of MAO-A is reduced in both variants.

**Figure 2:**
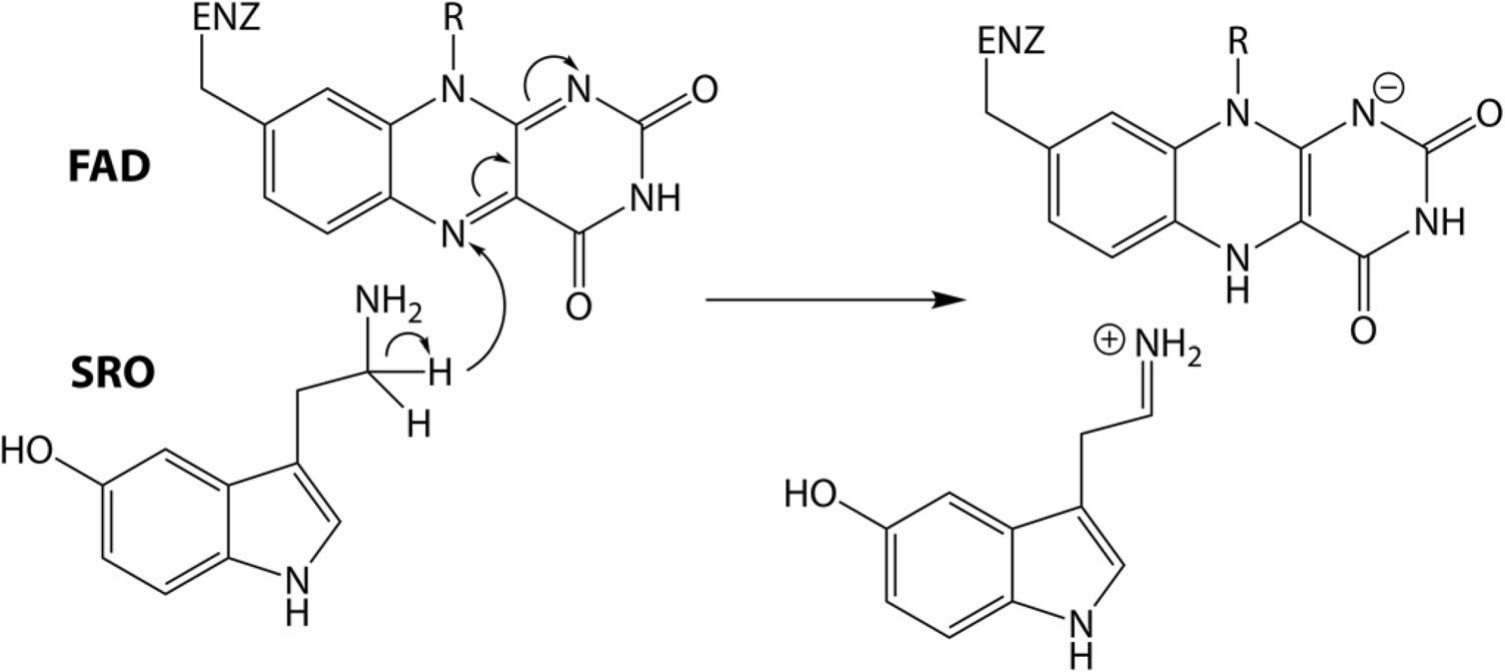
Scheme of the rate-limiting step of MAO-catalyzed serotonin degradation—hydride transfer between the reacting carbon atom of serotonin (SRO) vicinal to the amino group, to the nitrogen atom of the flavin adenine dinucleotide (FAD) cofactor inside the MAO active site.

From a chemical physics perspective, both R45W and E446K mutations of MAO-A provide a viable platform for an in-depth investigation of the possible alteration of the enzyme’s catalytic performance. Indeed, the replacement of a charged arginine residue by a neutral tryptophan (R45W) or negatively charged glutamate with positively charged lysine (E446K) has a significant influence on the electrostatic interactions not only in the vicinity of the mutated residue itself but also over a larger distance. Since the Coulombic interactions decrease linearly with distance, the change in a charge of a residue—whether from charged to neutral, or from negative to positive, or vice versa—may well affect the interactions with the reacting moiety, even if the mutated residue is located quite far (e.g., in the R45W variant over 18 Å) from the active site. In view of the hypothesis that enzyme catalysis is mainly governed by electrostatic interactions (i.e., by preorganized electrostatics)^6–10^, either mutation is the source of a substantial electrostatic perturbation that may severely affect the catalytic performance of MAO-A, probably impacting the metabolism of its substrates. In addition, the mechanism of the rate-limiting step of the oxidative decomposition of monoamine neurotransmitters (including serotonin) is well elucidated (Fig. 2)^11–15^, allowing for the construction of computational models that include not only a detailed description of the reacting moiety, but also the entire enzymatic environment that enhances reaction kinetics predominantly via electrostatics. Using the established Empirical Valence Bond (EVB) protocol, we were able to reproduce the catalytic performance of MAO enzymes at a quantitative level, specifically focusing on point mutation effects^16–18^, as well as substrate selectivity^19–21^. In addition, we devised our own multiscale model (*see below*) to demonstrate the critical role of electrostatics in the catalytic function of MAO-A^22^, and further expanded that model to elucidate the very subtle performance differences between the MAO-A and MAO-B isoenzymes^23^.

Probably the best experimental observable for enzyme electrostatics are electric fields exerted by enzymes onto their active sites^24^. However, measurement of these electric fields is not an effortless process and has been used to a limited extent—most notably, Boxer and co-workers have measured the electric field within the active site of ketosteroid isomerase^25^ using vibrational Stark effect spectroscopy^26^. Additional crystal structure analysis and computational modeling have shown that the electric field of ketosteroid isomerase is almost perfectly aligned to preferentially stabilize the transition state structure of its reaction^27^. There is wide-ranging scientific interest in the use of electric fields for catalysis, with many different experimental and computational studies both related to enzyme catalysis^25,27–30^ as well as more broadly^31–36^. Due to the limited use of experimental approaches connected to elucidating electric fields in enzymes and consequently the role of preorganized electrostatics in enzyme catalysis, the importance of computational approaches remains high in this case^37–44^.

Our own computational model^22,23^ allows us to test the role of electrostatics in the catalytic function of enzymes, thereby validating or disproving the hypothesis of preorganized electrostatics. Namely, we can evaluate the effect of electrostatics on the reaction rate by observing the influence of the electrostatic environment, expressed in the form of point charges, on several pertinent reaction parameters, particularly the activation barrier. Crucially for the purposes of investigating MAO-A mutants associated with the development of Brunner syndrome, an extension of our computational model allows us to elucidate the contributions of individual amino acid residues to the free energy barrier lowering. This means that we can compare the effects of these residues and observe the (potential) change in the barrier-lowering contribution when we mutate a wild-type residue to one associated with the clinical pathology of Brunner syndrome. In our recent study comparing the reactions of both MAO isoenzymes A and B through the lens of our computational model, we were able to discern even the very subtle differences between the two isoenzymes at the level of individual amino acid residue contributions and reproduce the experimental barrier difference of just 1.28 kcal mol^−1^ at quantitative precision^23^.

The goal of the present study was to acquire a clearer picture of the clinical manifestation of Brunner syndrome, a neuropsychiatric disorder connected to mutations in the *MAOA* gene sequence, by using several computational approaches to compare the catalytic performance of wild-type MAO-A with the R45W and E446K variants. At the same time, we continue our exploration of the role of preorganized electrostatics in enzyme (specifically MAO) catalysis, by applying our computational model to the MAO-catalyzed degradation of serotonin.

## Results

### Multiscale molecular dynamics simulation of the reactive step

**Figure 3:**
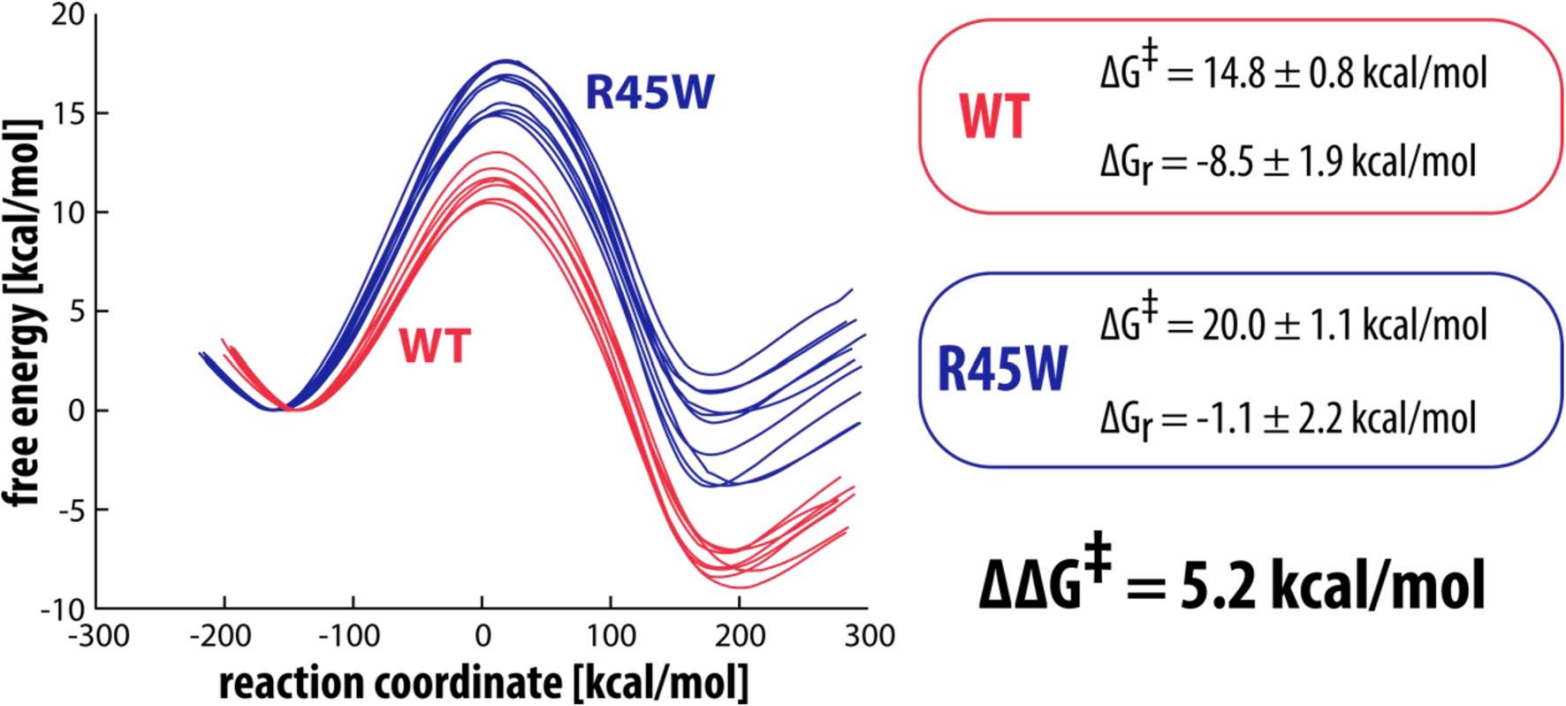
Free energy profiles for the rate-limiting step reaction between serotonin and the flavin cofactor in WT (red) and in R45W mutant (blue). The free energy barrier and the reaction free energy are also listed for both WT and R45W, with corresponding standard deviations.

**Figure 4:**
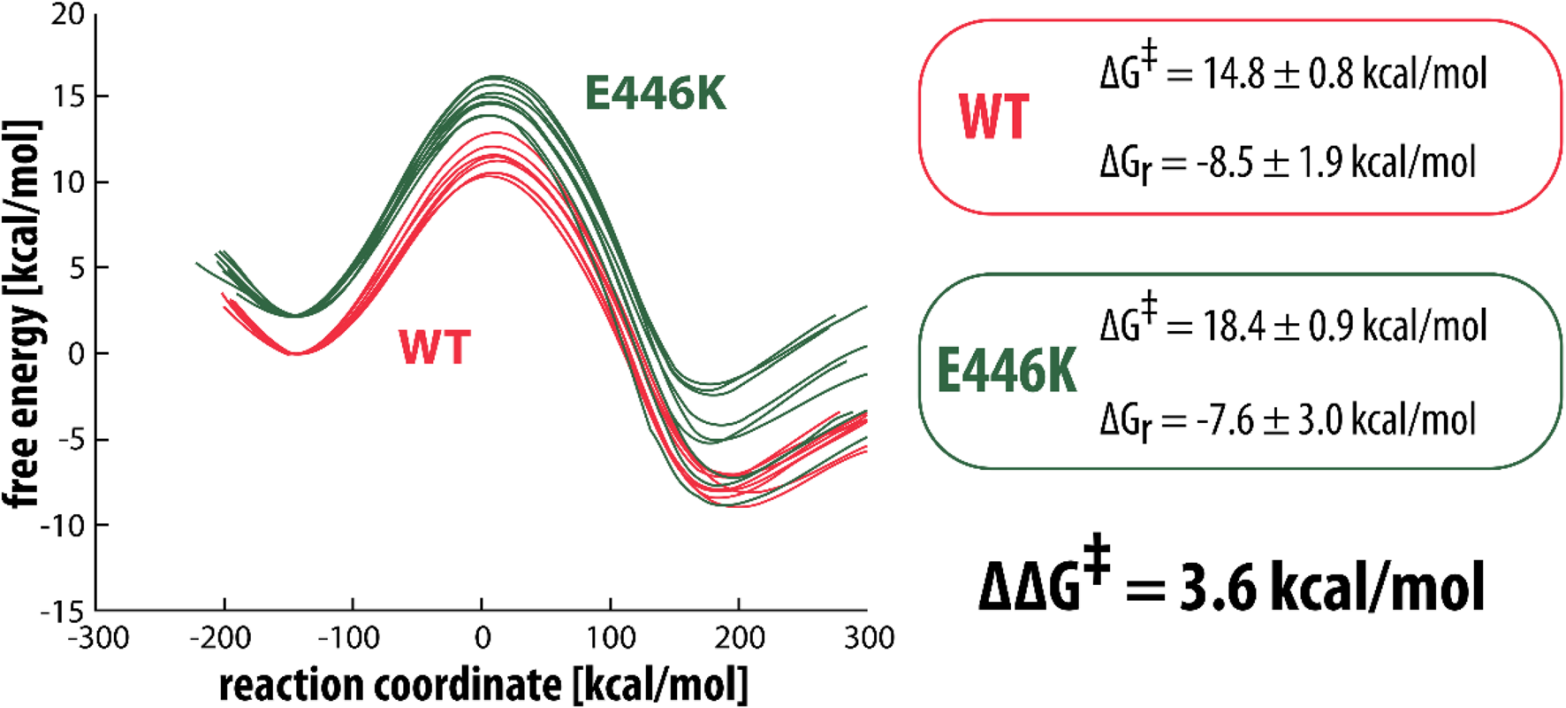
Free energy profiles for the rate-limiting step reaction between serotonin and the flavin cofactor in WT (red) and in E446K mutant (green). The free energy barrier and the reaction free energy are also listed for both WT and E446K, with corresponding standard deviations. In the E446K profiles deprotonation correction for Lys446 is included by considering the pKa value of Lys of 8.95, which at physiological conditions (pH = 7.4) converts into a free energy cost of 2.1 kcal/mol.

Free energy profiles for the reaction of serotonin with WT MAO-A and the mutated R45W and E446K variants are shown in Figs. 3 and 4, respectively. Values for the calculated free energy barriers (with the included correction for the free energy cost of serotonin deprotonation at a pH value of 7.4, calculated to be 3.6 kcal/mol^45^, and with the 2.1 kcal/mol correction for lysine deprotonation in the E446K variant) as well as the pertinent reaction free energies are also listed in Figs. 3 and 4. Using the established EVB methodology (described in more detail in Ref.^45^) we calculated a free energy barrier difference of 5.2 kcal/mol for R45W and 3.6 kcal/mol for E446K, which corresponds to about a 6000 and 450-fold decrease in enzymatic activity, respectively.

### Influence of electrostatic environment on reaction parameters

Using our own multiscale computational model, we are able to evaluate the influence of the presence of the electrostatic environment (electrostatics turned "ON" as compared to "OFF") on several quantities, namely the energy barrier (i.e., energy difference between the state of reactants and the transition state), charge transfer between the two reacting moieties, dipole moment and HOMO–LUMO gap (i.e., energy gap between the two reacting molecular orbitals). We find that for both WT MAO-A and the R45W mutant the presence of the electrostatic environment has a pronounced catalytic effect on all observed quantities (Table 1). Importantly, this catalytic effect is larger for WT than it is for R45W, which is in line with our EVB simulations (see Fig. 3) as well as with biochemical data.

**Table 1:** Effect of the presence (as compared to the absence) of the electrostatic environment of the enzyme on some parameters relevant for catalysis, calculated for WT MAO-A and the R45W mutant (standard error of the mean values are given in parentheses).

Quantity	Effect of electrostatics “ON” (relative to “OFF”)	Magnitude of effect	
		WT	R45W
Energy barrier	Decrease	14.0 (1.9) kcal/mol	13.1 (2.2) kcal/mol
Charge transfer	Increase	0.126 (0.016) *e*_0_	0.125 (0.012) *e*_0_
Dipole moment	Increase	0.39 (0.17) D	0.11 (0.23) D
HOMO–LUMO gap	Decrease	0.024 (0.002) a.u	0.019 (0.002) a.u

### Interplay between electric field and dipole moment

Additionally, we rationalized the effect of the electrostatic environment by observing the interaction between the electric field exerted by the enzyme and the dipole moment of the reacting moiety. We looked at the projections of both vectors onto the C…N axis (the C atom of serotonin and the N atom of the flavin cofactor) of the reacting moiety, which represents the presumed direction of electron flow. The product between the two vectors projected onto the C…N axis or the free energy of electrostatics ($G_{elec} \left( x \right)$) was evaluated, as described in the Computational Details section. All values are given in Table 2.

**Table 2:** The dipole moment of the reacting moiety and the magnitude of the electric field exerted by the enzymatic environment at the midpoint between the two reacting atoms (C of serotonin and N of lumiflavin) for the state of reactants (R) and for the transition state (TS).

	WT		R45W	
	R	TS	R	TS
Electric field (MV/cm)	− 35.0	− 61.9	− 24.2	− 44.7
Dipole moment (D)	3.9	− 0.7	5.0	0.1
$G_{elec} \left( x \right)$ (kcal/mol)	6.6	− 2.0	5.8	0.3
$\Delta G_{elec}^{\ddag } \left( x \right)$ (kcal/mol)	− 8.6	− 5.5		
$\Delta \Delta G_{elec}^{\ddag } \left( x \right)$ (kcal/mol)	3.1 (WT more catalytic than R45W)			

Both values are projected onto the C…N axis of the reacting moiety (presumed direction of electron flow). The value of the calculated free energy of electrostatics ($G_{elec} \left( x \right)$) is also given, as well as the difference in this quantity between TS and R. All values are given for WT and for R45W and the final difference in $\Delta G_{elec} \left( x \right)$ between the two is supplied.

For both WT MAO-A and the R45W mutant the electric field and the dipole moment change when going from R to TS. In both cases the magnitude of the electric field increases and also retains its direction (the sign remains negative). Interestingly, the direction of the dipole moment projection to the C…N axis changes when going from R to TS in the WT, but not in R45W. Therefore, while the interaction between the dipole moment and the electric field is switched from repulsive to attractive in the WT (going from R to TS), this is not the case with R45W, where this interaction remains repulsive in the TS. In R45W both R and TS are destabilized by the electrostatic environment (positive $G_{elec} \left( x \right)$ value), but the TS is destabilized to a smaller extent.

For both enzymes the final result is a barrier-lowering effect of the electrostatic environment, but this effect is more pronounced in WT (− 8.6 kcal/mol) than in R45W (− 5.5 kcal/mol). The calculated difference in $\Delta G_{elec}^{\ddag } \left( x \right)$ of 3.1 kcal/mol is in reasonable agreement with the free energy barrier difference calculated with EVB (5.2 kcal/mol). Because this analysis is a significant simplification of our system, we would not expect it to fully account for the total free energy barrier difference.

### By-residue analysis

The presented approach of depicting the electrostatic environment with an electric field vector and representing the reacting moiety with a dipole moment vector, allows us to partition the total barrier-lowering effect of an enzyme (i.e., $\Delta G_{elec}^{\ddag } \left( x \right)$) into contributions of specific amino acid residues. If we compare WT MAO-A to the R45W mutant all amino acid residues are of course the same except for Arg45 which is mutated into Trp45 in R45W.

Table 3 lists all residues for which the difference in $\Delta G_{elec}^{\ddag } \left( x \right)$ between WT and R45W is significant i.e., $\left| {\Delta \Delta G_{elec}^{\ddag } \left( x \right)} \right|$> 0.1 kcal/mol. This list consists of only 22 residues (out of a total of 512) with a significantly different composition as compared to the population of all residue pairs (Table 4). A lot of residues (13 out of 22) with $\left| {\Delta \Delta G_{elec}^{\ddag } \left( x \right)} \right|$> 0.1 kcal/mol are located very close (less than 10 Å) to the reacting moiety—in comparison, only 4% of all residues are located that close to the reacting moiety. On the other hand, only 4 out of 22 are located farther than 15 Å from the reacting moiety—one of these 4 is the mutated Arg45/Trp45 residue, which can be found roughly at a distance of 18 Å from the reacting moiety, the farthest out of all residues listed in Table 3. In WT MAO-A residue Arg45 has a catalytic effect (negative $\Delta G_{elec}^{\ddag } \left( x \right)$) of -0.93 kcal/mol. In mutated R45W residue Trp45 has a nearly vanished catalytic effect of − 0.04 kcal/mol, proving to be one of the bigger differences in the barrier-lowering effect of the electrostatic environment between WT MAO-A and R45W ($\Delta \Delta G_{elec}^{\ddag } \left( x \right)$ = 0.88 kcal/mol).

**Table 3:** Residues with biggest difference (between WT and R45W) in contribution to the barrier lowering effect due to the interaction between the electric field and the dipole moment.

Res. ID	Res. num	Dist. from origin (Å)	RMSD (Å)	$\Delta G_{elec}^{\ddag } \left( x \right)$ WT (kcal/mol)	$\Delta G_{elec}^{\ddag } \left( x \right)$ R45W (kcal/mol)	$\Delta \Delta G_{elec}^{\ddag } \left( x \right)$(R45W—WT) (kcal/mol)
GLN	215	8.0	1.0	0.93	− 0.55	− 1.48
GLY	66	7.4	1.3	0.43	− 0.64	− 1.08
ASN	181	8.8	2.3	− 0.13	− 1.05	− 0.92
TYR	69	7.3	1.0	0.33	− 0.45	− 0.77
TYR	444	6.6	0.7	− 0.21	− 0.70	− 0.49
ILE	180	9.8	2.6	− 0.33	− 0.64	− 0.31
GLY	443	8.0	0.5	0.21	0.00	− 0.21
GLU	436	14.0	0	− 0.05	− 0.21	− 0.16
MET	350	9.5	0.6	0.22	0.07	− 0.15
PHE	352	7.3	0.3	0.04	− 0.10	− 0.14
TYR	407	5.5	0.5	− 1.10	− 1.24	− 0.14
**490 amino acid residues with** $\left| {\Delta \Delta G_{elec}^{\ddag } \left( x \right)} \right|$< **0.1 kcal/mol**						
ARG	76	17.2	1.2	0.04	0.20	0.16
ARG	51	10.1	0.2	− 0.23	− 0.06	0.17
LYS	440	16.3	1.3	0.45	0.64	0.19
ARG	47	17.1	1.8	− 1.33	− 1.09	0.23
TYR	197	11.6	0.8	− 0.14	0.21	0.35
LYS	218	11.1	1.5	− 2.39	− 1.98	0.41
ASP	64	11.1	1.2	2.66	3.11	0.45
MET	445	8.6	1.6	− 0.55	− 0.07	0.47
**ARG/TRP**	**45**	**18.3**	**3.7**	− **0.93**	− **0.04**	**0.88**
ALA	68	6.3	3.8	− 1.72	0.12	1.84
GLY	67	5.7	3.0	− 1.47	0.80	2.27

The residues that have a larger catalytic effect in R45W are listed in the top half of the table (negative difference in $\Delta G_{elec}^{\ddag } \left( x \right)$) while the residues that have a larger catalytic effect in WT are listed in the bottom half of the table (positive difference in $\Delta G_{elec}^{\ddag } \left( x \right)$). Note that the total catalytic effect in R45W is by 3.1 kcal/mol smaller than in WT MAO-A. Average distance of residue from the midpoint between C of SRO and N of LFN is also given, as well as the RMSD between the average structures of WT and R45W. The mutated residue is marked in bold.

**Table 4:** Comparison of relative share of amino acid residues in some relevant groups in the total population of residue pairs (total of 512) vs. in pairs where $\left| {\Delta \Delta G_{elec}^{\ddag } \left( x \right)} \right|$> 0.1 kcal/mol (total of 22).

	Closer than 10 Å (%)	Farther than 15 Å (%)	Residue is charged (%)	RMSD > 2 Å (%)
All residue pairs (total of 512)	4	84	12	2
Pairs where $\left| {\Delta \Delta G_{elec}^{\ddag } \left( x \right)} \right|$> 0.1 kcal/mol (total of 22)	59	18	36	18

**Figure 5:**
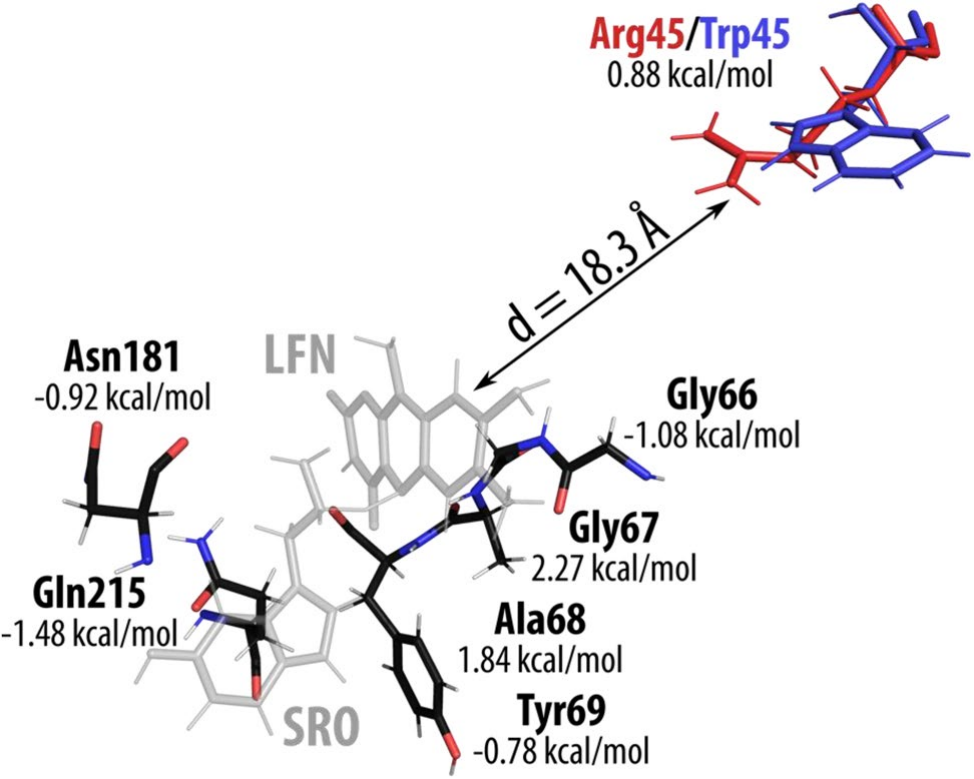
Residues with the largest difference in their barrier-lowering contribution between WT and R45W (i.e., $\left| {\Delta \Delta G_{elec}^{\ddag } \left( x \right)} \right|$> 0.5 kcal/mol)—a positive value of $\Delta \Delta G_{elec}^{\ddag } \left( x \right)$ indicates that the WT residue is more catalytic out of the two, while a negative value means the opposite. Reacting serotonin (SRO) and lumiflavin (LFN) are shown in grey, with the mutated residue (WT Arg45 in red and R45W Trp45 in blue) located at a distance of 18 Å from the reacting moiety.

Figure 5 shows those amino acid residues where $\left| {\Delta \Delta G_{elec}^{\ddag } \left( x \right)} \right|$> 0.5 kcal/mol. Apart from the mutated Arg45/Trp45 all other 6 residues are located close (less than 10 Å) to the reacting moiety. Both Gly67 and Ala68 have relatively large RMSD values (3.0 Å and 3.8 Å, respectively), meaning the 3D structure of both residues is significantly altered in R45W as compared to WT, which also influences their neighboring residues (Gly66 and Tyr69). Clearly, the observed structural differences are the consequence of structural relaxation invoked by the mutation, possibly extending beyond the nearest neighbors of the mutated residue, which is due to the highly complex network of non-bonding interactions between residues. Since the present mutation alters the charge of one residue, its effect is expected to be of quite a long range, because electrostatic interactions decay only linearly with the distance. Of course, this all affects the barrier-lowering contributions not only of the mutated residue, but also of the neighboring residues and farther beyond. Overall, a large RMSD indicating a bigger structural change between WT and R45W seems to be one of the more important factors affecting the barrier-lowering differences between the enzymes, with 4 out of 9 residues with RMSD > 2 Å providing a significant barrier-lowering difference ($\left| {\Delta \Delta G_{elec}^{\ddag } \left( x \right)} \right|$> 0.1 kcal/mol). The other five residues with RMSD > 2 Å are located far from the reacting moiety (19–25 Å) and do not have an important (anti-)catalytic effect on the barrier.

## Discussion

We investigated two mutated variants of MAO-A (namely p.R45W and p.E446K), which have been associated with the development of Brunner syndrome, a neuropsychiatric disorder characterized by intellectual disability and aggressive behavior. These symptoms in individuals affected by Brunner syndrome are thought to be related to elevated levels of serotonin, most probably connected to a decreased metabolism of this neurotransmitter associated with the impaired catalytic performance of the mutant. Our study focused on the rate-limiting step of serotonin decomposition catalyzed by MAO-A involving serotonin and the MAO cofactor FAD, comparing this reaction between WT MAO-A and the mutated R45W and E446K variants.

Our results show that the free energy barrier in both mutants is significantly higher than in WT MAO-A, namely by 5.2 kcal/mol in R45W and by 3.6 kcal/mol in E446K, which in both cases corresponds to a significant (6000 and 450-fold, respectively) decrease in the decomposition rate (which is for practical purposes identical to a *MAOA* knockout) and would suggest a drastic increase in serotonin levels. This is in line with clinical data on Brunner syndrome and especially with observations on the herein considered variants, where serotonin levels are significantly elevated, and the metabolism of this neurotransmitter is apparently disproportionally affected. This is in a way similar to results of studies done on the Y444F MAO-A mutant, where the mutant retained a reasonable level of catalytic efficiency in benzylamine metabolism, while oxidation of serotonin was 120-fold slower than in the WT^46^.

Additionally, we elucidated the role of the enzyme's electrostatic environment on catalysis in both WT MAO-A and R45W using our own computational model. We found that the presence of the electrostatic environment has a significant catalytic effect on all observed parameters and, crucially, that this catalytic effect is more pronounced in WT MAO-A than it is in R45W. Further analysis of dipole moment and electric field interaction shows a destabilizing interaction in the state of reactants for both WT and R45W MAO-A. But while this interaction changes to stabilizing in the transition state for the WT, it stays destabilizing in R45W. The total barrier lowering difference between WT MAO-A and R45W was calculated to be 3.1 kcal/mol (WT more catalytic than R45W), which is in reasonably good agreement with the EVB calculated barrier difference of 5.2 kcal/mol. Additional by-residue analysis shows that larger structural differences (bigger RMSD) between the enzymes correspond to larger differences in the barrier-lowering contributions of individual amino acid residues, especially if these residues are located close to the reacting moiety. Even so, the mutated residue (arginine in WT, tryptophan in R45W) which is located at a distance of 18 Å from the reacting moiety is one of the biggest contributors to the difference between the enzymes.

The results presented herein provide valuable new information about two of the mutant versions of MAO-A associated with the development of Brunner syndrome by confirming the decreased rate of serotonin metabolism compared with WT. We anticipate a similar trend for the remaining point mutations, leading to the clinical observation that Brunner syndrome is genetically heterogeneous. It remains a major challenge for the future to address kinetic data for all clinically relevant mutants experimentally and computationally.

It should be noted that, to date, all studies that have addressed Brunner syndrome have focused mainly on investigating changes in (plasma or urinary) metabolite levels caused by various mutations in the *MAOA* gene sequence. No in-depth kinetic or mechanistic studies have been performed. A possible reason for the lack of experimental kinetic data are time-consuming and expensive experimental procedures. Therefore the present study fills an important gap in our previous knowledge of Brunner syndrome while providing a link between chemical physics, genetics and clinical picture. Moreover, the important role of electrostatics in enzyme catalysis is also confirmed for the MAO-catalyzed degradation of serotonin.

## Methods

### Molecular dynamics simulations

The model of MAO-A used in this study was built on the basis of the crystal structure of the enzyme deposited in the Protein Data Bank (ID code 2Z5X)^47^, prepared as described in our previous study of the wild type-catalyzed degradation of serotonin (SRO)^45^. For the purposes of this study, in the R45W variant the Arg45 residue was changed to Trp45, whereas in E446K Glu446 was changed to Lys446 using the UCSF Chimera program^48^. All other conditions stayed the same as in our wild type study. The simulation model was built using the OPLS-AA force field^49^ and consisted of a spherical cell with a radius of 30 Å and centered on the *N*5 atom of the flavin cofactor, encompassing the enzyme–substrate complex and 1649 water molecules. The ligand parameters were acquired by the ffld_server utility and assisted by the Maestro v. 11.7 graphical interface^50^. The atomic charges of the ligand were computed by fitting to the electrostatic potential calculated at the HF/6-31G(d) level of theory according to the RESP scheme, as implemented in AmberTools18^51^.

The system was first relaxed and equilibrated in several steps, by gradually increasing the time step (from 0.1 to 1 fs) and the temperature (from 1 to 300 K) as well as diminishing the positional restraints. From this equilibrated structure, ten distinct replicas were generated, and standard Empirical Valence Bond (EVB) procedure^52–54^ based on the Free Energy Perturbation/Umbrella Sampling approach^55,56^ was employed for the simulation of the reactive step. The force field of the reactants ($\varepsilon_{1}$) was transformed into the force field of the products ($\varepsilon_{2}$) in 51 subsequent mapping steps via the coupling parameter $\lambda$ using a mapping potential of the type:

$$\varepsilon_{m} = \lambda \varepsilon_{1} + \left( {1 - \lambda } \right)\varepsilon_{2}$$.

Each mapping step was 10 ps long, yielding a total of 5.1 ns of MD. The free energy profiles were determined using the same EVB parameters that we obtained in the wild type study (the off-diagonal matrix element *H*_*ij*_ of 44.28 kcal mol^−1^ and the gas-phase shift *α* of 103.94 kcal mol^−1^), by fitting to the quantum chemically computed reaction parameters (the barrier height and the reaction energy) for the gas phase. All simulations were performed using the *Q5* program package^57^ and visualization of trajectories was done using the *VMD* program package^58^.

### Electrostatics analysis

Analysis of the electrostatic environment of both the wild type MAO-A and the R45W variant was done using our own computational model, described in detail in our previous studies^22,23^. For this analysis we treated the reacting moiety (SRO and the truncated flavin cofactor, lumiflavin—abbrev. LFN) with the quantum treatment at the M06-2X/6–31 + G(d,p) level, while the rest of the solvated enzyme was represented only with point charges. This kind of representation ensures that the only interaction between the reacting moiety and its environment comes from Coulombic forces. Therefore, by performing quantum computations in the presence as well as in the absence of the point charges, we can evaluate the influence of the electrostatic environment on several parameters we consider to be important for catalysis (namely the energy barrier between the state of reactants and the transition state, charge transfer between serotonin and the truncated flavin cofactor, the dipole moment, and the energy gap between the two reacting molecular orbitals i.e., the HOMO–LUMO gap). For this approach we require ‘snapshots’ (i.e., characteristic structures) of our system in the state of reactants (R) and in the transition state (TS), as those are the two states relevant for evaluating catalysis. For the R45W variant, these snapshots were acquired from the above-described MD simulation, while for the wild type they were obtained from the simulation described in Ref.^45^. For each simulation, we extracted 100 snapshots for both R and TS, giving us 400 in total. For all snapshots the energy and the electronic structure of our system were computed in the presence and in the absence of 13,193 point charges corresponding to the surrounding protein and water molecules. The electronic structure was analyzed using the Natural Bond Orbital v. 3.1 method^59^. Analysis of the HOMO–LUMO gap was done by separately treating SRO and LFN in the same point charge surroundings. The HOMO of SRO was taken directly from the quantum computation, while the LUMO of LFN was approximated as the negative of the electronic affinity. All quantum computations were performed using the *Gaussian09* program package^60^.

Additionally, the electric field ($\vec{\varepsilon }$) at the midpoint between the C of SRO and the N of LFN was evaluated for all snapshots, by using the following fundamental expression:

$$\vec{\varepsilon } = \mathop \sum \limits_{i} \frac{{q_{i} }}{{4\pi \epsilon_{0} r_{i}^{2} }}\left( {\frac{{\overrightarrow {{r_{i} }} }}{{r_{i} }}} \right),$$

where $\epsilon_{0}$ is the free space permittivity constant, $q_{i}$ is the charge of the *i*-th atom, $r_{i}$ is the distance of the *i*-th atom from the point of interest (i.e., the midpoint between the C of SRO and the N of LFN), and $\overrightarrow {{r_{i} }}$ is the vector connecting the *i*-th atom and the point of interest. The dot product of the electric field vector and the dipole moment vector yields the free energy of the electrostatic interaction between the reacting moiety and the surrounding enzyme ($G_{elec}$). The difference in this value between R and TS gives the barrier change due to the (de)stabilizing interaction between the dipole moment and electric field vectors:

$$\Delta G_{elec}^{\ddag } = - \left( {\vec{\varepsilon }_{TS} \cdot \vec{\mu }_{TS} - \vec{\varepsilon }_{R} \cdot \vec{\mu }_{R} } \right).$$

Note that the negative sign in the above expression comes from the opposite orientation definitions for the electric field and the dipole moment vectors—their parallel alignment results in stabilization (negative $G_{elec}$), while their antiparallel alignment results in destabilization (positive $G_{elec}$). When the electric field is given in MV/cm and the dipole moment in Debyes, the above expression needs to be multiplied by a factor of 0.048 to give the result in kcal/mol. This approach also allows us to analyze $\Delta G_{elec}^{\ddag }$ in terms of contributions of individual amino acid residues.

## Data Availability

The structure of wild-type MAO A used in the present research is available in the RCSB Protein Data Bank database (https://www.rcsb.org/, accession code 2Z5X). Other data generated during and/or analyzed in the current study are available from the corresponding author on reasonable request.
